# An extensive database on the traits and occurrences of amphibian species in Turkey

**DOI:** 10.1038/s41597-024-03101-w

**Published:** 2024-03-14

**Authors:** Dilara Arslan, Burak Akdağ, Çağdaş Yaşar, Anthony Olivier, Yanina Benedetti, Federico Morelli, Kerim Çiçek

**Affiliations:** 1 Czech University of Life Sciences Prague, Faculty of Environmental Sciences, Kamýcká 129, CZ-165 00, Prague, 6 Czech Republic; 2 Section of Zoology, Department of Biology, Faculty of Science, Ege University, Izmir, Turkey; 3 Tour du Valat, Institut de Recherche pour la Conservation des Zones Humides Méditerranéennes, Le Sambuc, 13200 Arles, France; 4 Natural History Application and Research Centre, Ege University, Izmir, Turkey

**Keywords:** Biodiversity, Biogeography, Herpetology

## Abstract

Amphibians are the most endangered taxa among vertebrates, and they face many threats during their complex life cycles. The species’ life history traits and occurrence database help understand species responses against ecological factors. Consequently, the species-level-trait database has gained more prominence in recent years as a useful tool for understanding the dimensions of communities, assembly processes of communities, and conserving biodiversity at the ecosystem level against environmental changes. However, in Turkey, there are deficiencies in the knowledge of the ecological traits of amphibians compared to other vertebrate taxa, as most studies have focused on their distribution or taxonomic status. Consequently, there is a need to create such a database for future research on all known extant amphibians in Turkey. We compiled a species-level data set of species traits and occurrences for all amphibians in Turkey using 436 literature sources. We completed 36 trait categories with 5611 occurrence data for 37 amphibian species in Turkey. This study provides an open, useful, and comprehensive database for macroecological and conservation studies on amphibians in Turkey.

## Background & Summary

The biodiversity crisis is particularly evident in amphibians and is significantly higher than in other vertebrates^1,2^. One in three of the world’s 8,000 known amphibian species is threatened with extinction, and nearly half are in decline^1,3^. Understanding the factors affecting amphibian species is essential for predicting species extinction risk and effectively planning their conservation^4^. This requires that species traits and occurrence data be evaluated together concerning their interactions with biotic-abiotic factors in their ecosystems^5^.

The species-specific traits studies increasingly gain attention from ecologists to determine species distribution and community composition or make conservation efforts more effective^5–7^. This is due to the strong relationship between species-specific traits and the organism’s functionality and suitability for its environment. For instance, life history traits data are needed to answer a wide range of questions in evolution, ecology, and conservation biology, especially for amphibians. Amphibians’ survival relies on sustaining highly specialized environmental conditions due to their ectothermic physiology and complex life cycle^8,9^. Considered to be highly influenced species in the external environment, they can be restricted to a narrow distribution, while more generalist ones can reach more extensive distributions^10–12^. Recent publications have combined amphibians’ ecological traits to create comprehensive open databases for ecological and evolutionary research^13–15^. However, they contain very limited data for the amphibians of Turkey and show low coverage. Among these, Oliveira *et al*.^14^ assessed 78% of the species in Turkey, but matrix completeness is quite low for species in Turkey; for example, 81% of species were not assessed for seasonality, 71% for feeding, or 11% for habitat type. In another study by Trochet *et al*.^13^, only 15% of the amphibian species in Turkey were assessed in European List. Huang *et al*.^16^ evaluated less than half of Turkey’s amphibians in a list of only morphological traits database^16^. Apart from this, amphibians species traits may vary according to geographical location differences, even if they are the same taxa^17^, and wider scale assessments are more prone to error^5^.

The scientific study of Turkish herpetofauna began in the 19th century with the emergence of natural history exploration and the establishment of museums and research institutions^18^. European naturalists and explorers contributed to documenting and classifying amphibian species in Turkey^19–21^. Over the last 50 years, numerous studies have been carried out in various regions of Turkey, shedding light on the distribution and abundance of amphibians^22^. These studies contributed to developing field guides, taxonomic revisions, and conservation efforts focused on protecting endangered species and their habitats, e.g.^18,23–26^. Turkey is home to 37 species of amphibians which include two orders: Caudata (newts and salamanders) and Anura (toads and frogs)^18^. Among amphibians species in Turkey, 30% of amphibian species (11 species) are listed as threatened, 10 of which are among the 13 species endemic to the country^18^. However, there is a lack of ecological studies of amphibians in Turkey compared to other vertebrate taxa. Most existing studies are concerned with their distribution or taxonomic status. In this study, we aim to complete the ecological traits of amphibians for future ecological studies in Turkey. Considering its uniqueness, we collected two database releases of information on Turkey amphibians from 436 compiled literature sources. We made them freely accessible as 36 species traits and an extensive list of occurrence data for 37 amphibians’ species.

## Methods

### Taxonomic list

We followed^12,18^ as a taxonomic reference for listing Turkish batrachofauna. This list comprises 37 amphibian species, with 20 caudatans and 17 anurans.

### Databases

To create a comprehensive trait and occurrence dataset for amphibians, we incorporated the database categories in alignment with the classifications presented in Trochet *et al*.^13^, Oliveira *et al*.^14^, Wells^8^, and Neves *et al*.^15^. The species traits and occurrence databases consist of extensive literature reviews. The research of literature for these two databases was initially conducted through a systematic review of peer-reviewed scientific publications accessed online through academic platforms: Web of Science (https://www.webofscience.com/), Vipers Garden (http://vipersgarden.at/lit_db.php), Council of Higher Education database of Turkey (https://tez.yok.gov.tr/UlusalTezMerkezi/), Google Scholar (https://scholar.google.com/). Data were also collected from field guidebooks or books^8,22–25,27–29^, specialized websites (e.g., amphibiaweb.org), and grey literature (e.g., technical reports, government documents, monographs, and dissertations). The literature search was conducted using the following keywords and their different combinations: species’ Latin names, synonym names, and ‘English and Turkish names’, ‘Turkey’, ‘Türkiye’, ‘Anatolia’, ‘Anadolu’, ‘Thrace’, ‘Trakya’, ‘Asia Minor’, ‘Herpetofauna’, ‘Amphibia’, ‘Anura’, ‘Urodela’, ‘Salamander’, ‘Caudata’ and city names in Turkey.

### Trait data base

We identified 36 traits related to species morphology (1), life history traits (2), habitat preferences and distribution (3), and threats (4) for each species— each trait evaluation is expressed in Supplementary Table 1. For all quantitative trait values in morphometric and life history traits, we only considered publications presenting individuals from Turkey. More than one existing source for the quantitative values in morphometric and life history traits was averaged by weighing the mean of each species in the reference study according to the sample size. All morphometric values were presented with weighted SD and sample size values (N). The DD (lack of data) values in the database mean no data has been reported for the relevant traits in the literature. The NE (Not Evaluated) values in the database mean this trait has not been evaluated for the relevant species.

Morphometry traits (1) were evaluated in 5 subcategories traits: Snout Vent Length (SVL), Total Body Length (TBL), Foreleg Length (FLL), Hind Leg Length (HLL), and Tibia Length (TL.). TBL was not evaluated for anurans and T.L. for urodeles. All values are given in millimeters (mm), with minimum and maximum values for each species’ females, males, and adults, and mean values for adults only. Unlike Trochet *et al*.^13^, we didn’t include total body weight because the weight measurement varies between the species identified in the museum that ethanol-preserved animals and those measured from live specimens (McDiarmid 1994)^30^. The life history traits (2) category was evaluated within the 19 subcategories of traits representing: consist with reproduction, development, food, communication behavior, and movement traits when available in the reference sources. The habitat preferences and distribution traits category (3) consists of two databases one is categorical, and the second one is spatial data (spatial data are in Supplementary Table 1, tab named as “occurrence database”). The first trait table of habitat preferences and distribution was evaluated within the five subcategory traits (habitat type, topography, landscape type, Turkey biogeographical region, and E.U. biogeographical region). Amphibians’ habitats need to be explained locally, in landscapes, and regionally for better management^31^; therefore, we evaluated the habitats of amphibians in these three levels. We assessed local habitat characteristics by classifying habitat type and topography, which emphasizes which part of the landscape species can inhabit. For the landscape level, we use CORINE (2018) habitat classification to assess what kind of surrounding habitats the species can survive in. For the regional level, we assessed the biogeographical levels in Turkey and the E.U. for each species. Finally, we followed IUCN threat assessments (Version 2023-1) in the threat traits (4) and ended with 3 subcategories of traits (IUCN threat category, Population trends, and major threats)^32^. The definition of all traits is listed in “MetaData” tab and their value in “Trait Database” tab in “xlsx”. format in Supplementary Table 1. All evaluated publications are presented in “References List” tab in Supplementary Table 1 (the full reference list).

### Occurrence database

This database is the last updated scientific literature collection up to February 2023 on Turkish herpetofauna. It includes various databases, including museum collections of scientific and grey literature (Supplementary Table 1). Species occurrences data are classified according to accuracy in 2 classes: literature, and Google Earth (Google Inc. ver. 9.3.). The records obtained from literature were classified into two accuracy classes: “Literature” and “Google Earth”. Localities for which coordinated data are available in the literature were also used directly, and their accuracy was classified as “Literature”. Locality information without coordinate data in the literature was referenced using Google Earth (Google Inc. ver. 9.3.) according to the location definitions in the publications. Then their accuracy was classified as “Google Earth.” All records were georeferenced to the WGS-84 coordinate system, checked, and visualized with ArcGIS Pro (ESRI). All geographical coordinates in “xlsx”. format are presented in “Occurrence Database” tab in Supplementary Table 1.

## Data Records

**Fig. 1:**
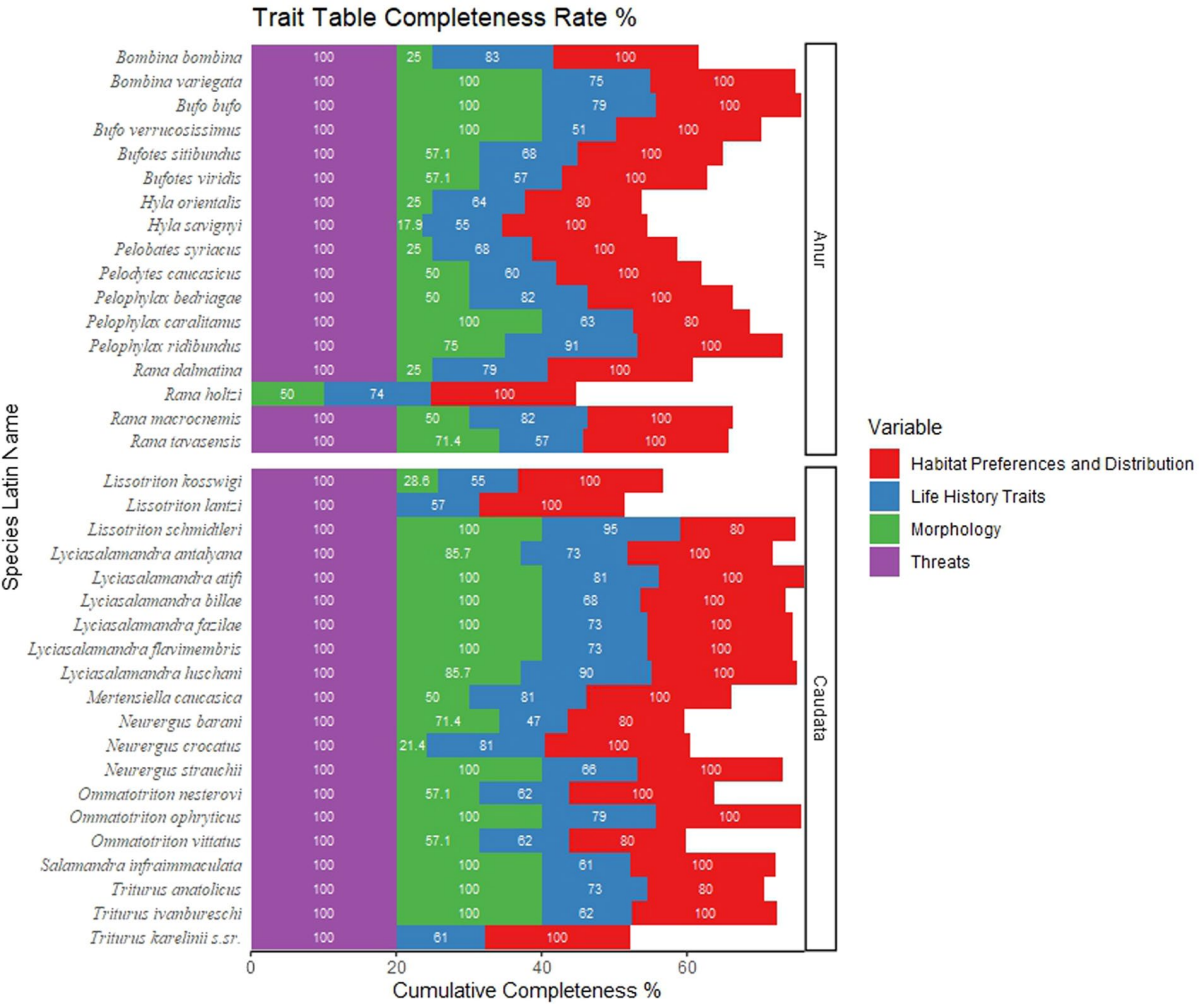
The database completeness (%) for Amphibians of Turkey.

**Fig. 2:**
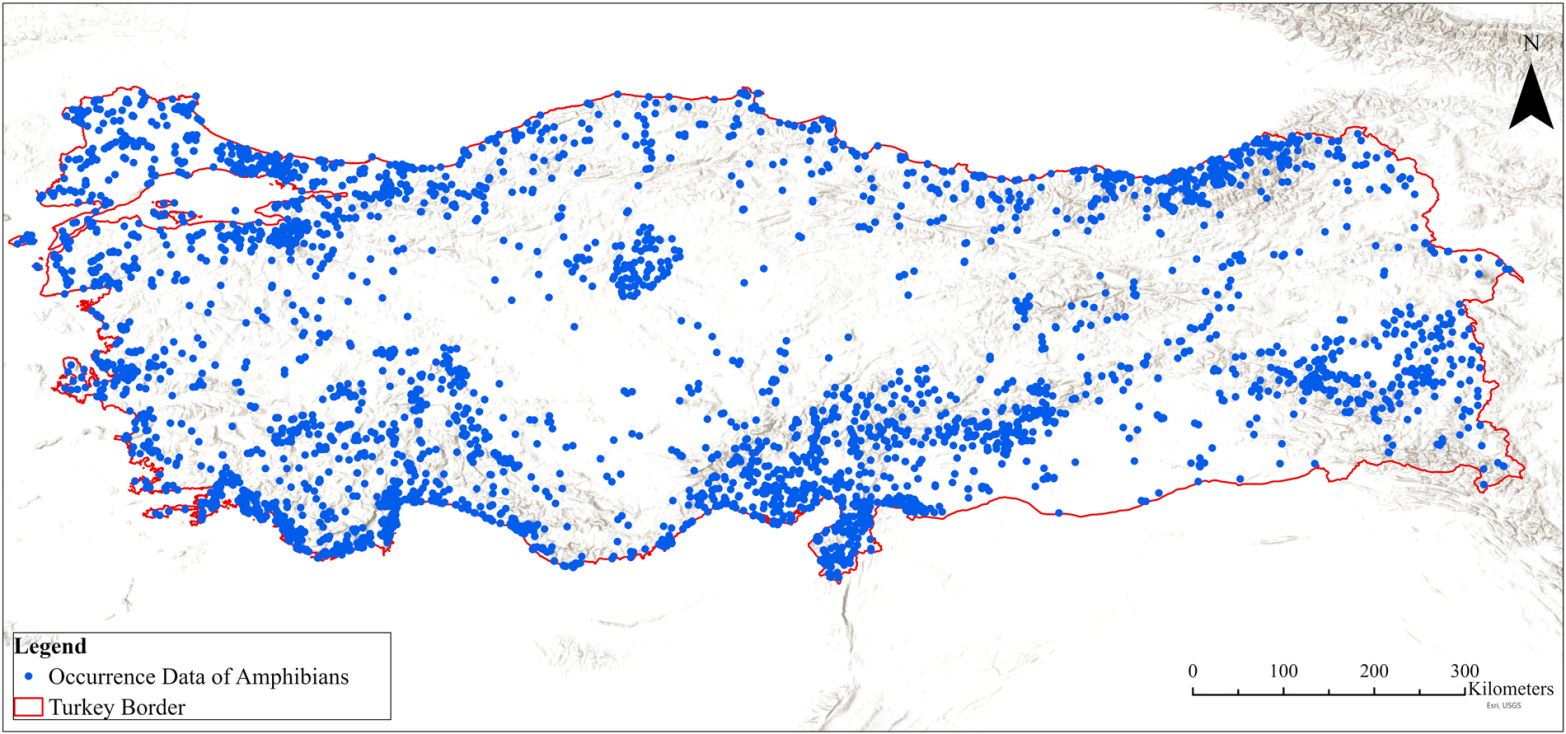
Occurrence data of all amphibians in Turkey. Blue dots show the locations.

A total of 436 literature sources were used to evaluate 37 species’ traits (269 different sources) and occurrences (215 different sources) (Supplementary File 1). The amphibian’s trait and occurrences databases can be downloaded from Figshare^33^ as a single Excel file (in “.xlsx” format) with multiple tabs. The overall completeness rate for the trait table is 82.4%. The completeness rate for each trait category is as follows: 65.8% for morphology traits, 72.3% for life history traits, 96.7% for habitat preferences and distribution, and 97.2% for threats (Fig. 1). It should be noted that the completeness of the table is very limited for 2 species: *L. lantzi* and *T. karelinii s.l*., these species are at the edge of their range and therefore the amount of research on these species in Turkey is very limited^18,34^. Finally, we provided 5611 occurrence records data for 37 species (4272 for anurans, and 1339 for urodeles). It should be noted here that the information that there is an old museum record of Fire Salamander (*Salamandra salamandra*) and Common spadefoot (*Pelobates fuscus*) occurring in western Turkey has not been evaluated in this study because its existence is not known for certain^18^. There is also a recent study by Yaşar *et al*.^18^ on the possible distribution of Balkan spadefoot (*P. balcanicus*) on the Greek-Turkish border in northwestern Turkey. However, there is still some doubt about *P. balcanicus* distribution in Turkey, so it has not been included in the list due to the need for confirmation^18^. We should emphasize that our distribution database contains limited occurrence data in Central Anatolia (Fig. 2). In this region, we have obtained a very limited number of amphibian occurrence data compared to other parts of the country: only 8% of all occurrence data is from this region (Fig. 2).

## Technical Validation

All data set was obtained manually from the reference source for each species and carefully recorded. Double control procedures were applied by the authors (DA, BA, and KÇ) to ensure the dataset was extracted from the relative sources. The data were then re-examined and validated by other authors. During the entire control procedures, the trait dataset was controlled for outliers or abnormal values by using boxplots. Then, controlled the source to see how this measurement was taken; if there is no explanation, we accepted those values as outliners. In a few where inconsistencies arose, the decision was based on the authors independently cross-checking the original sources twice and reaching a consensus through mutual agreement. Besides, the occurrence dataset was controlled for georeferencing errors (e.g. points in the ocean or out of known distribution zone).

## Usage Notes

Our study provides for the first time an accessible data set for the amphibians of Turkey with this trait and a spatial occurrence database. All database releases are available on *figshare* (Data Repository)^33^ in a single .xlsx file with multiple tabs that include the defined tabs in following:

Supplementary Table 1

Trait Database

Occurrence Database

Trait Database Refences List

MetaData

References List

### Supplementary information

Supplementary Information

## Data Availability

No custom code was used to generate the described databases.
