# Supplementary material for: An extensive database on the traits and occurrences of amphibian species in Turkey
Source: Sci Data. 2024 Mar 14;11:292. doi: 10.1038/s41597-024-03101-w (PMC10940290; doi:10.1038/s41597-024-03101-w)
Supplement: Supplementary file 1 — Supplementary Information [file 41597_2024_3101_MOESM1_ESM.docx]

### Supplementary Information

**Table S1.** The number of references used for each species. TDRID: Trait Database Reference ID; # of TDR: Total number of references in the trait database; ODRID: Occurrence Database Reference ID; # of ODR: Total number of references in the occurrence database.

| **Species** | **TDRID** | **# of TDR** | **ODRID** | **# of ODR** |
| --- | --- | --- | --- | --- |
| Bombina bombina | 28, 52, 54, 55, 72, 91, 97, 112, 166, 130, 307, 333, 346, 347, 353, 392, 366 | 17 | 17, 72, 97, 111, 112, 176, 233,307,370, 403, 427, 429 | 12 |
| Bombina variegata | 28, 31, 52, 54, 55, 72, 91, 100, 101, 126, 136, 166, 181, 213, 316, 333, 347, 366, 392 | 19 | 99, 100, 233 | 3 |
| Bufo bufo | 28, 52, 54, 55, 72, 91, 145, 147, 149, 151, 184, 214, 298, 307, 347, 366, 392, 166, 409 | 19 | 11, 20, 145, 147, 149, 170, 172, 186, 188, 203, 225, 228, 237, 243, 249, 250, 251, 259, 273, 276, 300, 399, 402, 426, 431 | 25 |
| Bufo verrucosissimus | 28, 52, 54, 55, 72, 91, 145, 146, 166, 298, 347, 355, 392 | 13 | 6, 17, 137, 145, 163, 172, 186, 221, 259, 276, 300, 403 | 13 |
| Bufotes sitibundus | 26, 28, 52, 54, 55, 72, 91, 144, 166, 179, 185, 347, 392, 405 | 14 | 2, 9, 12, 16, 17, 23, 25, 26, 30, 60, 64, 86, 87, 90, 103, 137, 143, 144, 149, 161, 170, 177, 179, 186, 188, 203, 209, 210, 215, 230, 237, 238, 247, 249, 251, 271, 273, 276, 301, 332, 360, 363, 385, 399, 402, 403, 404, 420, 426, 427 | 51 |
| Bufotes viridis | 28, 52, 54, 55, 72, 91, 149, 151, 160, 166, 254, 256, 315, 329, 340, 342, 347, 354, 392, 437, 439 | 21 | 11, 143, 186, 301, 363, 403, 427 | 7 |
| Hyla orientalis | 8, 24, 28, 52, 54, 55, 72, 91, 156, 166, 299, 334, 347, 351, 392, 400 | 16 | 3, 4, 6, 11, 42, 87, 112, 142, 163, 170, 177, 186, 188, 201, 203, 215, 217, 230, 234, 237, 239, 249, 250, 251, 271, 352, 367, 376, 399, 403, 406, 427, 430, 436 | 35 |
| Hyla savignyi | 18, 19, 28, 52, 54, 55, 72, 91, 140, 166, 226, 347, 350, 392, 415 | 15 | 9, 16, 86, 172, 186, 201, 273, 332, 352, 402, 406, 415, 420, 426, 436 | 15 |
| Lissotriton kosswigi | 18, 28, 52, 54, 55, 72, 88, 91, 139, 162, 166, 325, 347, 348, 392 | 15 | 344, 396, 397 | 3 |
| Lissotriton lantzi | 28, 52, 54, 55, 72, 91, 166, 240, 325, 343, 347, 348, 392, 438 | 14 | 396 | 1 |
| Lissotriton schmidtleri | 1, 28, 29, 52, 54, 55, 72, 79, 89, 91, 118, 128, 133, 134, 139, 166, 193, 307, 321, 325, 347, 348, 375, 392, 427 | 25 | 42, 133, 186, 193, 344, 396, 397 | 7 |
| Lyciasalamandra antalyana | 13, 28, 52, 54, 55, 72, 74, 91, 166, 194, 198, 294, 304, 305, 319, 324, 347, 348, 386, 392, 416 | 21 | 13, 186, 293, 339, 359, 391, 435 | 7 |
| Lyciasalamandra atifi | 15, 27, 28, 52, 54, 55, 72, 91, 166, 195, 280, 281, 282, 294, 304, 319, 324, 341, 348, 347, 362, 392, 417, 422 | 24 | 186, 195, 293, 359, 387, 391, 435 | 7 |
| Lyciasalamandra billae | 28, 52, 54, 55, 72, 91, 96, 166, 182, 190, 192, 194, 195, 197, 294, 304, 319, 324, 341, 347, 348, 392 | 22 | 186, 190, 197, 435 | 4 |
| Lyciasalamandra fazilae | 28, 52, 54, 55, 72, 75, 91, 166, 189, 194, 195, 303, 304, 318, 319, 324, 341, 347, 348, 383, 392, 417 | 22 | 186, 195, 293, 359, 367, 387, 391, 435 | 8 |
| Lyciasalamandra flavimembris | 28, 41, 43, 52, 54, 55, 72, 91, 166, 191, 304, 319, 324, 338, 347, 348, 381, 392 | 18 | 186, 191, 195, 293, 381, 387, 391, 435 | 8 |
| Lyciasalamandra luschani | 14, 28, 52, 53, 54, 55, 72, 73, 75, 81, 91, 166, 195, 231, 283, 294, 304, 319, 324, 341, 347, 348, 392, 416 | 24 | 14, 72, 186, 195, 293, 359, 387, 391, 435 | 9 |
| Mertensiella caucasica | 28, 50, 52, 54, 55, 72, 80, 91, 164, 166, 260, 347, 348, 356, 378, 392 | 16 | 80, 163, 186, 202, 251, 359, 410 | 7 |
| Neurergus barani | 28, 52, 54, 55, 72, 85, 91, 166, 279, 287, 292, 302, 347, 348, 382, 392 | 16 | 84, 186, 253, 287, 302 | 5 |
| Neurergus crocatus | 28, 52, 54, 55, 72, 91, 166, 274, 275, 302, 307, 336, 347, 348, 349, 373, 392 | 17 | 58, 159, 302 | 3 |
| Neurergus strauchii | 28, 52, 54, 55, 72, 91, 122, 166, 287, 288, 302, 307, 328, 347, 348, 382, 392, 401, | 18 | 16, 46, 84, 86, 253, 287, 302, 404, 421 | 9 |
| Ommatotriton nesterovi | 22, 28, 52, 54, 55, 71, 72, 91, 95, 151, 166, 205, 255, 264, 284, 296, 322, 347, 348, 380, 386, 392 | 22 | 45, 59, 61, 72, 95, 186, 215, 251, 271, 335, 369, 386, 430 | 13 |
| Ommatotriton ophryticus | 22, 28, 52, 54, 55, 65, 71, 72, 83, 91, 95, 166, 180, 255, 284, 286, 296, 307, 322, 347, 348, 380, 386, 392 | 24 | 59, 72, 95, 186, 285, 380, 386, 403, 430 | 9 |
| Ommatotriton vittatus | 22, 28, 52, 54, 55, 72, 83, 91, 95, 166, 290, 307, 348, 347, 386, 392 | 16 | 51, 72, 86, 95, 117, 186, 332, 367, 380, 386, 420, 426 | 12 |
| Pelobates syriacus | 28, 52, 54, 55, 72, 91, 98, 108, 131, 134, 135, 139, 166, 180, 224, 294, 307, 326, 347, 366, 370, 372, 392, 413 | 24 | 2, 28, 42, 72, 82, 83, , 129, 170, 177, 186, 203, 224, 248, 361, 367, 369, 371, 385, 403, 408, 418, 424, 426, 427, 430 | 25 |
| Pelodytes caucasicus | 28, 38, 52, 54, 55, 72, 91, 113, 165, 166, 174, 183, 206, 216, 261, 267, 307, 346, 347, 364, 392, 411 | 22 | 3, 6, 17, 28, 34, 63, 72, 163, 165,172, 186, 200, 216, 222, 262, 412 | 16 |
| Pelophylax bedriagae | 28, 52, 54, 55, 67, 70, 72, 77, 91, 119, 123, 138, 154, 166, 204, 257, 294, 297, 347, 366, 392, 439, 440 | 23 | 10, 42, 78, 109, 119, 138, 150, 186, 188, 203, 215, 220, 239, 250, 273, 297, 317, 332, 337, 399, 403, 420, 426 | 23 |
| Pelophylax caralitanus | 28, 32, 39, 52, 54, 55, 69, 72, 91, 92, 160, 166, 168, 171, 207, 307, 347, 365, 392 | 19 | 10, 17, 37, 39, 66, 87, 92, 150, 161, 168, 171, , 177, 186, 207, 235, 242, 367 | 17 |
| Pelophylax ridibundus | 28, 35, 47, 48, 49, 52, 54, 55, 72, 78, 91, 120, 126, 151, 166, 169, 175, 204, 246, 257, 278, 312, 314, 320, 345, 347, 354, 392, 433 | 29 | 2, 4, 6, 9, 10, 11, 12, 16, 30, 33, 46, 47, 52, 60, 78, 86, 94, 102, 103, 105, 109, 112, 114, 127, 132, 137, 149, 163, 170, 177, 186, 188, 203, 217, 230, 237, 245, 249, 251, 252, 308, 309, 317, 332, 337, 339, 358, 368, 369, 402, 403, 404, 420, 426, 427, 432 | 56 |
| Rana dalmatina | 28, 52, 54, 55, 72, 91, 157, 166, 187, 199, 211, 323, 331, 414, 347, 366, 389, 392, 414 | 19 | 30, 54, 57, 64, 72, 157, 172, 186, 215, 217, 237, 249, 251, 271, 369, 403, 414, 427, 430 | 19 |
| Rana holtzi | 28, 44, 52, 54, 55, 72, 83, 91, 166, 199, 270, 347, 392, 411, 419, 423 | 16 | 61, 68, 72, 110, 235, 272, 306, 388, 389, 403, 411, 434 | 12 |
| Rana macrocnemis | 5, 28, 40, 44, 52, 54, 55, 56, 72, 91, 115, 116, 124, 166, 218, 256, 258, 268, 307, 313, 327, 347, 368, 392 | 24 | 3, 4, 5, 6, 12, 16, 17, 46, 86, 127, 137, 163, 170, 172, 177, 186, 188, 212, 223, 227, 243, 249, 250, 251, 272, 330, 332, 358, 368, 389, 399, 402, 404, 425 | 34 |
| Rana tavasensis | 28, 36, 52, 54, 55, 56, 72, 91, 106, 107, 126, 152, 166, 223, 240, 294, 327, 347, 348, 389, 392 | 21 | 72, 107, 152, 186, 388, 403 | 6 |
| Salamandra infraimmaculata | 21, 28, 52, 54, 55, 72, 83, 91, 104, 121, 166, 229, 288, 291, 307, 347, 348, 390, 392 | 19 | 7, 16, 46, 86, 104, 186, 288, 332, 359, 384, 391, 402, 404, 426 | 14 |
| Triturus anatolicus | 28, 50, 52, 54, 55, 72, 91, 93, 166, 269, 284, 285, 289, 311, 325, 347, 348, 357, 379, 392, 398 | 21 | 186, 251, 310, 311, 395 | 5 |
| Triturus ivanbureschi | 28, 50, 52, 54, 55, 72, 91, 158, 166, 193, 219, 265, 266, 289, 295, 325, 347, 348, 357, 392, 394, 395, 427, 428 | 24 | 28, 42, 72, 155, 177, 186, 193, 215, 217, 232, 244, 247, 250, 393 | 14 |
| Triturus karelinii | 28, 50, 52, 54, 55, 72, 91, 166, 241,263, 325, 347, 348, 357, 377, 392 | 16 | 403 | 1 |
